# Immune Modulatory Effects of Vitamin D on Herpesvirus Infections

**DOI:** 10.3390/ijms26041767

**Published:** 2025-02-19

**Authors:** Daniel Galdo-Torres, Sabina Andreu, Oliver Caballero, Israel Hernández-Ruiz, Inés Ripa, Raquel Bello-Morales, José Antonio López-Guerrero

**Affiliations:** Departamento de Biología Molecular, Universidad Autónoma de Madrid, 28049 Madrid, Spain; daniel.galdo@uam.es (D.G.-T.); oliver.caballero@estudiante.uam.es (O.C.); ines.ripa@uam.es (I.R.); ja.lopez@uam.es (J.A.L.-G.)

**Keywords:** vitamin D, immune modulation, herpesvirus, cathelicidin (LL-37), herpes simplex virus 1, herpes simplex virus 2, cytomegalovirus, Epstein–Barr virus

## Abstract

In addition to its classical role in calcium and phosphate metabolism regulation, vitamin D also has an important impact on immunity modulation. Vitamin D regulates the immune response, shifting from a proinflammatory state to a more tolerogenic one by increasing the release of anti-inflammatory cytokines while downregulating proinflammatory cytokines. Thus, low levels of vitamin D have been associated with an increased risk of developing autoimmune diseases like multiple sclerosis and type 1 diabetes. Furthermore, this prohormone also enhances the release of well-known antimicrobial peptides, like cathelicidin LL-37 and β-defensins; therefore, it has been proposed that vitamin D serum levels might be related to the risk of well-known pathogen infections, including herpesviruses. These are a group of widely spread viral pathogens that can cause severe encephalitis or tumors like Kaposi’s sarcoma and Burkitt lymphoma. However, there is no consensus on the minimum levels of vitamin D or the recommended daily dose, making it difficult to establish a possible association between these two factors. This narrative non-systematic review will analyze the mechanisms by which vitamin D regulates the immune system and recent studies about whether there is an association between vitamin D serum levels and herpesvirus infections.

## 1. Introduction

Vitamin D is a secosteroid prohormone mainly obtained through the exposure of the skin to ultraviolet B (UVB) radiation. There are two major forms of vitamin D: vitamin D2 (ergocalciferol) and vitamin D3 (cholecalciferol). Vitamin D2 is synthesized from ergosterol and can be found in mushrooms and plants. On the other hand, vitamin D3 is produced endogenously in the skin from the cholesterol precursor 7-dehydrocholesterol when exposed to sunlight, which is the main source of vitamin D for humans [1]. However, some foods, such as oily fish and eggs, can also serve as direct sources of this prohormone [2]. In the liver, cholecalciferol and ergocalciferol are converted by hydroxylation to 25-hydroxyvitamin D (calcifediol, 25(OH)D), an intermediate inactive form, which is then converted to 1,25-dihydroxyvitamin D (calcitriol), the active form of vitamin D, by cytochrome P27B1 (CYP27B1) (also known as 25-hydroxyvitamin D 1-alpha-hydroxylase) in the kidney and other tissues. This metabolite is responsible for vitamin D signaling through the nuclear receptor vitamin D receptor (VDR), modulating gene expression.

Once calcitriol activates the VDR, the ligand–receptor complex is dimerized with the retinoic X receptor (RXR), followed by translocation into the nucleus and attachment to Vitamin D Responsive Elements, thereby modifying gene expression [2,3,4]. VDRs can cycle in and out of the nucleus and can also bind to DNA in a ligand-independent manner; however, most known actions of VDR require its ligand [5]. They possess a nuclear localization signal (NLS) that is recognized by RXRs if it is intact. The presence of 1,25(OH) D enhances this process and ensures efficient nuclear transport regardless of the NLS status [6].

**Figure 1:**
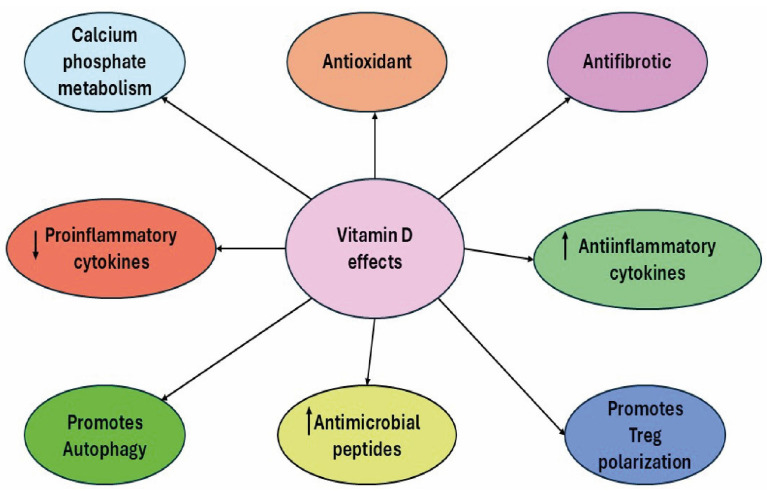
Effects of vitamin D on human beings. Despite vitamin D’s classical role in calcium and phosphate metabolism regulation, it also has other vital impacts on the human body, including antioxidant and antifibrotic effects, the promotion of autophagy and Treg polarization, increasing the number of antimicrobial peptides released, decreasing proinflammatory cytokines, and promoting anti-inflammatory cytokine liberation.

Vitamin D is well-known for its role in the regulation of calcium and phosphate metabolism [7,8]. In addition to this, vitamin D also has an important immunomodulatory effect on the innate and adaptative immune systems, as well as antioxidant and anti-fibrotic properties [2,3,9,10] (Figure 1). This is supported by the presence of VDR and the metabolization of enzymes in well-known immune cells like macrophages, monocytes, lymphocytes, and dendritic cells [11]. Therefore, vitamin D might play a key role in autoimmune diseases and infections by maintaining self-tolerance [12,13] and decreasing the release of proinflammatory cytokines such as IL-1, IL-6, and TGF-β [8,13]. The effects of vitamin D on vascular permeability [14], rheumatoid arthritis [15], psoriasis [16,17], and hepatitis B infections [18,19] have already been demonstrated by a vast number of publications.

The *Herpesviridae* family is a group of enveloped, double-stranded DNA viruses with the ability to infect a wide variety of species, including humans. To date, of the more than 200 recognized herpesvirus members, 8 are known to primarily infect humans: herpes simplex virus 1, herpes simplex virus 2, varicella zoster virus, Epstein–Barr virus, cytomegalovirus, human herpesvirus 6, human herpesvirus 7, and human herpesvirus 8 [20]. This family is divided into three subfamilies: *Alphaherpesvirinae*, *Betaherpesvirinae*, and *Gammaherpesvirinae* [21,22]. Herpesvirus infection can be divided into three distinct phases: acute infection, latency, and reactivation. Acute infection takes place in epithelial cells, where the virus replicates and generates a substantial number of virions. This phase is usually asymptomatic or associated with mild symptoms. After primary replication, the virus spreads to other regions of the organism to establish latency, during which no virion production occurs. Finally, if the immune system is compromised, the virus can reactivate its replicative cycle and restart virion formation, which is associated with several feasible manifestations in various tissues [22,23,24]. However, despite being one of the most prevalent viral families in the world, the number of studies on the effects of vitamin D during herpesvirus reactivations and their outcomes is limited.

The definition and significance of vitamin D deficiency remain topics of ongoing debate. The aim of this narrative non-systematic review is to study the possible association between vitamin D serum levels and herpesvirus infections and reactivations. To accomplish this, we will first study the vitamin D statuses based on vitamin D serum levels (deficiency, insufficiency, and adequate) and the different criteria for classification. Following that, we will review the currently known immunomodulatory effects of vitamin D in viral infections. Subsequently, to better identify a possible association, we will analyze both in vitro and in vivo published studies, including observational studies, cohort and case–control studies, and controlled trials with treatment or placebo. Regarding the studies involving the human population, the infection or reactivation of human herpesvirus will be defined by clinical or laboratory criteria.

## 2. Vitamin D Status: Discrepancies in Minimum Levels and Global Deficiency

Vitamin D levels are defined as the circulating concentration of total serum 25(OH)D (calcifediol) levels, since the active form 1,25-(OH)_2_D_3_ (calcitriol) has a short half-life and depends on calcium homeostasis [3]. There is no current consensus on the exact minimum levels. The lack of an exact minimum desirable concentration also means that the recommended daily doses of vitamin D vary widely (200 IU to 2000 IU). This variability is also present in therapeutic regimens, with differences in the administration frequency (daily, weekly, or monthly) and formulations, with both oral and intramuscular formulations available [25,26,27,28]. However, many guidelines agree that serum 25(OH)D levels below 25 or 30 nmol/L (10–12 ng/mL) should be avoided at any age since this is the minimum level that protects against vitamin D-related bone disease [28,29]. For instance, the Clinical Guidelines Subcommittee of The Endocrine Society defines vitamin D deficiency as serum levels of 25(OH)D below 20 ng/mL (50 nmol/L), while the US Institute of Medicine lowers the threshold to 12 ng/mL (30 nmol/L) [30,31]. Current studies consider sufficient levels of vitamin D to be above 40 ng/mL, while optimal levels are around 50 ng/mL [25,26,32].

Vitamin D deficiency is a widespread global health issue. In terms of prevalence, a recent study involving a cohort of 7.9 million participants reported that about 47.9% of the global population has serum vitamin D levels below 20 ng/mL, while 15.7% have severe deficiency (12 ng/mL) [33]. In the European population, a study by Cashman et al. showed that over 40% of people have vitamin D insufficiency (<20 ng/mL), while 13% have vitamin D deficiency (<12 ng/mL) [34]. Surprisingly, a similar prevalence of vitamin D deficiency is found in low-latitude countries with high sunlight exposure. In South America, 34.67% of the population has vitamin D serum levels below 20 ng/mL [35]. This deficiency can be attributed to a combination of lifestyle, environmental, biological, and cultural factors. As UVB is the main source of vitamin D, insufficient sunlight exposure (high-latitude countries, indoor lifestyles) contributes to deficiency [33], as do diets low in vitamin D, skin pigmentation, and age-related factors [1,36,37].

The lack of vitamin D has been associated with an increased risk of chronic diseases in adults, such as autoimmune diseases [15], osteopenia (higher risk of fractures), multiple sclerosis (MS), cardiovascular disease, and type 1 and 2 diabetes, among others [38]. In children, a deficiency may retard growth and hinder the achievement of normal bone mineral density [39], leading to skeletal deformities. Conversely, excessive supplementation can lead to adverse effects, such as hypercalcemia, with symptoms such as vomiting, muscle weakness, and kidney damage [40].

## 3. Vitamin D and Viral Infections

### 3.1. Vitamin D Boosts Cathelicidin LL-37 Synthesis

One of the ways in which vitamin D modulates the immune response in the presence of infection is through the synthesis of cathelicidin LL-37, an antimicrobial peptide that plays a key role in innate immunity against infections [4,41,42,43]. Calcitriol can access monocytes and macrophages via passive diffusion or scavenger receptors (SR) like SR-B1 and enhances the synthesis of cathelicidins via VDR-RXR signaling [44].

Cathelicidins are host defense peptides widely distributed in animals. In humans, only one cathelicidin has been characterized, named LL-37. These peptides are stored in a pre-pro-peptide form in different granules, from which they are released upon activation. Once released, the pre-pro-peptide is cleaved to its mature form [45]. Mature cathelicidins share a common cationic charge and hydrophobic residues organized in α-helical or β-sheet structures. These characteristics result in antimicrobial activity since the positive charge can disrupt Gram-positive and Gram-negative bacteria membranes, as well as viral envelopes [45,46]. Furthermore, the positive charge can interact with negatively charged molecules such as RNA or DNA, stimulating the uptake of these molecules and the activation of toll-like receptors (TLR), leading to an increase in cytokine expression and nitric oxide production [47].

**Figure 2:**
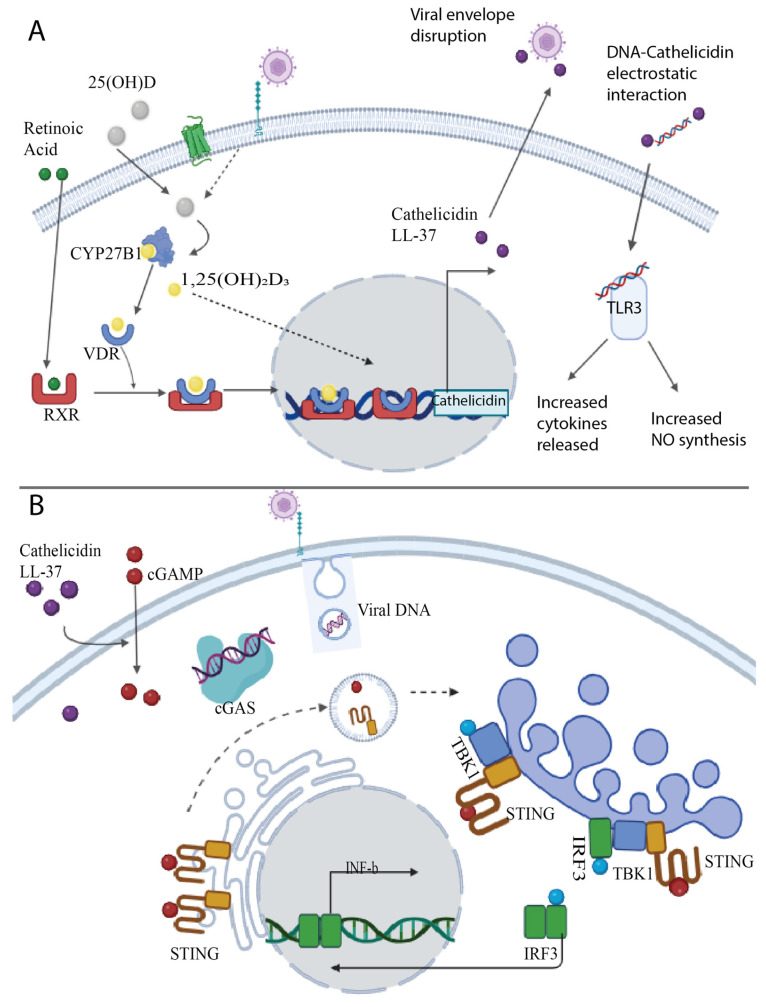
Vitamin D enhances the release of antimicrobial peptides. (**A**) Viral recognition enhances CYP27B1 enzyme activity, which converts 25(OH)D to 1,25(OH)D or calcitriol. Once the VDR is activated by calcitriol, the ligand–receptor complex is dimerized with the RXR, followed by translocation into the nucleus and attachment to Vitamin D Responsive Elements, increasing cathelicidin release. Lower levels of VDR can interact with DNA in the absence of its ligand. (**B**) cGAS acts as a cytosolic sensor of DNA. When viral DNA is detected, cGAS starts to produce cGAMP, a secondary messenger that binds to STING. This triggers STING oligomerization. Subsequently, STING recruits TABK1, through which phosphorylation mediates the recruitment of IRF3. Then, IRF3 is dimerized and translocated to the nucleus to regulate gene expression and enhance the release of cathelicidin LL-37. Cathelicidin LL-37 promotes the entry of cGAMP into the target cell, spreading the antiviral response to other cells. Based on [48].

In addition to this, LL-37 can enhance type I interferon (IFN) production in macrophages and monocytes via STING signaling, inducing a refractory viral state. In this cellular pathway, cyclic 2′-3′-GMP-AMP (cGAMP) synthase (cGAS) acts as a cytosolic sensor for DNA and tissue damage. During viral infection, this enzyme recognizes viral DNA and synthesizes cGAMP, a secondary messenger that binds to STING. This binding leads to well-known conformational changes that trigger STING oligomerization and activation. Subsequently, STING recruits TANK-binding kinase 1 (TBK1), through which phosphorylation mediates the recruitment of Interferon Regulatory Factor 3 (IRF3). IRF3 is then dimerized and translocated to the nucleus to regulate gene expression [48]. cGAMP is also responsible for spreading and amplifying the refractory viral state to neighboring cells in a paracrine manner, enhancing innate immunity. Since cGAMP is both anionic and hydrophilic, the presence of a transporter is required for its entry through the cell membrane. It has been proposed that LL-37 can mediate the entrance of cGAMP into the cell, therefore providing a significant role in antiviral immunity [49]. Figure 2 shows a schematic representation of the antiviral activity of cathelicidins.

Furthermore, cathelicidins can stimulate phagocytosis [50] and degranulation in mast cells [51], thereby increasing the immune response against different pathogens. The antiviral activity of cathelicidins has already been evaluated against a wide range of viruses, such as rhinoviruses [52,53], coronaviruses [54], and herpesviruses [43,46,55].

### 3.2. Vitamin D and Other Innate Immunity Mechanisms

On one hand, vitamin D can stimulate the production of other antimicrobial compounds by Paneth cells and epithelial cells. Among the antimicrobial cocktails secreted by these cells, defensins and lysozymes are the main components. Both exhibit antibacterial and antiviral activity [56,57]. The most well-known mechanism of action of these peptides is lipid perturbation. However, several studies have shown that these peptides are also active against non-enveloped viruses through other mechanisms. For enveloped viruses, such as herpesviruses, the main ‘antiviral’ mechanism involves the alteration of glycoproteins involved in receptor binding. On the other hand, for non-enveloped viruses, defensins bind to the viral capsid, allowing entry into the cell while altering intracellular trafficking, thus compromising the viral cycle [58]. Lysozymes are also cationic peptides with antiviral activity. Their positive charges enable them to interact with viral DNA, neutralizing its negative charge and altering its conformation, thereby compromising viral replication and transcription. In addition, it has been reported that lysozymes can also interact with RNA polymerase, interfering with viral replication [59].

In addition to its activity in enhancing other antimicrobial compounds, vitamin D also regulates specific cell line activation. In the presence of an infection, high levels of proinflammatory cytokines and IFN-γ induce the activation of TLR1/TLR2, enhancing the expression of CYP27B1 and VDR and increasing calcitriol levels in macrophages. TLR activation also promotes the generation of IL-15 and IL-1β, amplifying the response through a loop mechanism. This leads to increased cathelicidin and β-defensin synthesis, as well as the recruitment of the autophagy and inflammasome systems to support pathogen eradication [2,11]. However, to avoid detrimental effects on the host, high levels of calcitriol in monocytes eventually down-regulate the expression of TLR2/TLR4/TLR9, reducing IL-6 and IFN-γ production and shifting to a more tolerogenic state [11,60].

Furthermore, vitamin D can also stimulate invariant natural killer T cells (iNKT), a group of immune cells derived from the conventional double-positive T cell stage [61]. This type of cell provides protection against pulmonary viral infections by increasing specific CD8+ T cells and antibodies against viruses. In addition, the release of INF-γ by these cells can enhance NK cell and macrophage activation, both of which are essential for the control of viral infections [62,63].

### 3.3. Vitamin D and Adaptative Immunity

Regarding adaptative immunity, calcitriol secreted by monocytes and macrophages enhances the shift of the immune system from a proinflammatory to a tolerogenic state. Calcitriol promotes the differentiation of T-helper lymphocytes (Th) from a Th1 and Th17 to a Th2 profile, increasing the release of anti-inflammatory cytokines such as IL-4 and IL-5, while decreasing IL-2 and IFN-γ levels. Moreover, calcitriol promotes differentiation into regulatory T cells (Treg), enhancing the anti-inflammatory response [8]. This is very useful in viral infections in which the release of proinflammatory cytokines is out of control, resulting in cytokine storm syndrome, as seen in COVID-19. This syndrome contributes to the induction of inflammatory cell death and tissue damage, exacerbating the symptoms [64,65].

In addition, vitamin D has been identified as a modulator of several viral infections, such as rhinovirus, Dengue, and Severe Acute Respiratory Syndrome Coronavirus 2 (SARS-CoV-2), through more specific mechanisms [66,67,68,69,70].

## 4. Immunomodulatory Activity of Vitamin D in Herpesvirus Infection

Thanks to its immunomodulatory activity [42,46,55,71,72], it is interesting to study a possible association with widespread viruses, such as herpesviruses.

### 4.1. Alphaherpesvirus

**Figure 3:**
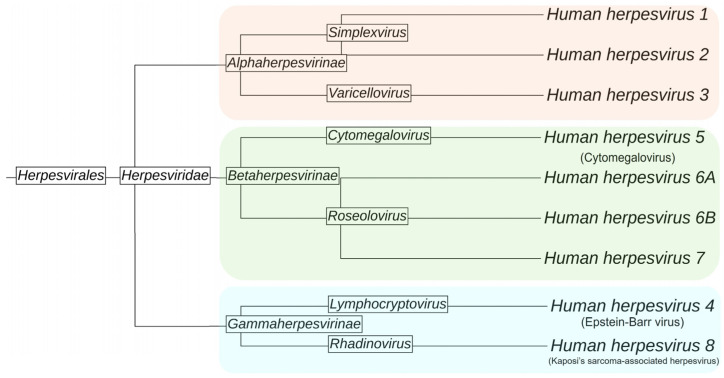
Phylogenetic tree of human herpesviruses. Viral subfamilies *alpha*, *beta*, and *gammaherpesviridae* are highlighted in red, green, and blue, respectively. Figure made with iTOL (v.6) [77].

The *Alphaherpesvirinae* subfamily encompasses neurotropic viruses that, after causing epithelial lesions, disseminate into the sensory ganglia or the peripheral nervous system, establishing latency in ganglia soma. Within the alphaherpesviruses, there are viruses with a significant impact on human health (Figure 3), such as herpes simplex virus 1 (HSV-1 or HHV-1) [73], herpes simplex virus 2 (HSV-2 or HHV-2), and varicella zoster virus (VZV or HHV-3), as well as relevant viruses in animal health, like canine herpes virus 1 (CHV-1) and pseudorabies virus (PRV) [74]. According to the World Health Organization (WHO), around 70% of the adult population is infected with HSV-1, while 13% is infected with HSV-2. Although most primary infections are self-limited, in some cases, the infection may lead to severe symptoms like encephalitis or herpetic keratitis [21,75,76].

#### 4.1.1. In Vitro Studies

As in many other viral infections, the cGAS-STING pathway is essential for the immune response [22]. Thus, since vitamin D can boost this pathway by increasing cathelicidin LL-37 synthesis, it may play a pivotal role in controlling herpesvirus infections. Previous in vitro assays have shown the antiviral activity of cathelicidins and cathelicidin-derived peptides against different alphaherpesviruses like PRV, HSV-1, and HSV-2 [43,46,55]. LL-37 has been reported to exhibit antiviral properties in many cell lines, such as human corneal epithelial cells, by preventing HSV-1 from binding to the cells [78], as well as in Vero cells derived from the kidney of an African green monkey [79], and preventing HSV-2 infection in Vero cells, keratinocytes, and skin biopsies from mice [79,80]. Regarding vitamin D’s effect on HSV-1 in vitro, a notable reduction in viral titers was reported in infected HeLa cells that were treated with either 25(OH)D_3_ or 1,25(OH)_2_D_3_ [81].

#### 4.1.2. Studies in the Human Population

Studies involving humans analyzing the association between vitamin D and HSV-1 and HSV-2 infections are scarce due to the difficulty of diagnosis. In addition, no studies regarding this direct association in animal models have been performed to date. A cross-sectional study conducted in pediatric-onset multiple sclerosis (MS) showed a weakly positive relationship between serum vitamin D levels and HSV-2 titers in MS patients, although this correlation was not found in the control group. No evidence of an association between vitamin D serum levels and HSV-1 antibody titers in either group was found. However, the sample size of this study was too small, and a causal association cannot be assumed. Furthermore, it must be taken into account that MS patients are usually immunocompromised, and their antibody titers must be considered critically [82]. In addition, the presence of comorbidities may impact the immune system’s ability to respond to vitamin D supplementation. However, the direct impact of these comorbidities on vitamin D’s effectiveness against herpesviruses is not yet well-studied in the studies mentioned.

Paradoxically, Parvaie et al. found a negative association between vitamin D deficiency and the presence of HSV-1, although this relationship was not significant, as the sample population was too small (100 people), suggesting inconclusive results [83].

On the other hand, a recent study involving more than 14,000 individuals revealed a positive correlation between vitamin D deficiency and HSV-1 or HSV-2 seroprevalence in people [84]. Furthermore, a significant association between low serum vitamin D levels and the presence of recurrent herpes labialis (RHL) was also observed [85]. In addition, Ranjbar et al. observed that lower levels of vitamin D were, indeed, related to a longer healing duration of the lesions in RHL, although they did not find an association between vitamin D serum levels and a positive history of RHL [86]. However, none of these studies evaluated the effects of vitamin D supplementation, and it remains unclear whether it is protective. Given these data, no clear evidence of the protective effects of vitamin D supplements in herpes simplex infections has been observed, and further studies are needed.

Regarding VZV infection, there is still some discrepancy as to whether serum vitamin D levels are related to the incidence of VZV reactivation. A study from the National Taiwan University Hospital compared VZV-IgG and VZV-IgM titers in chronic dialysis patients with insufficient or sufficient serum vitamin D concentrations. Their results showed differences in VZV-IgG serum levels based on serum vitamin D concentrations, although no significant differences in VZV-IgM titers were observed. Patients with 25(OH)D levels greater than 27.8 ng/mL had significantly higher VZV-IgG levels than those with insufficient or deficient levels, indicating that higher serum 25(OH)D levels correlate with greater immunity [87].

On the contrary, a more recent study conducted in the general population in the United Kingdom claims that there is no relationship between low vitamin D levels and a major risk of VZV reactivation, even though vitamin D supplementation may decrease VZV among hemodialysis patients. Nor was an association found between vitamin D supplementation and VZV reactivation, although it is worth noting that the data on vitamin D supplementation in this study were based on a self-reported survey, rather than being assigned in a double-blind randomized controlled trial, as in similar studies [88].

These discrepancies may be due to differences between the population studies, although the first study only included chronic renal-ill patients, who are generally immunocompromised, while the second study focused on the general population, which is largely immunocompetent. Also, both studies used different classifications of vitamin D insufficiency or deficiency. Overall, the benefits of vitamin D supplementation in preventing VZV reactivation remain unclear. All the alphaherpesvirus studies considered for this review and their characteristics are described in Table 1.

### 4.2. Betaherpesvirus

Among the *Betaherpesvirinae*, human cytomegalovirus (HCMV or HHV-5) is one of the most relevant and studied viruses. According to the Centers for Disease Control and Prevention (CDC), half of adults worldwide have been infected with this virus by the age of 40, while one in three children are infected by the age of 5 [89]. HCMV can infect most cells in the human body, and it is transmitted through saliva, blood, or even urine. Despite causing mainly asymptomatic disease in immunocompetent populations, immunocompromised individuals can develop serious clinical manifestations. This is especially relevant during pregnancy or after transplantation since blood transfusions and organ transplants could be a means of spreading the infection. Moreover, active HCMV infection is a predictor of acute rejection in transplantations, and HCMV infection is one of the most common congenital infections worldwide [21,90].

#### 4.2.1. In Vitro Studies

A recent in vitro study demonstrated that HCMV can induce vitamin D resistance in mammalian cells through the downregulation of VDR [91]. Thus, this could be a mechanism of immune evasion by HCMV. According to Stecher et al., this resistance to vitamin D is due to the upregulation of the transcriptional repressor Snail1. The expression of this repressor is significantly increased upon transcription of the immediate early genes (IE1 and IE2) of HCMV. This study also revealed that cathelicidin LL-37 treatment of the infected cells can significantly reduce the viral titers of HCMV at non-cytotoxic concentrations. These results might indicate that VDR downregulation is an important immune evasion mechanism of HCMV and that vitamin D supplementation may enhance the immune response in HCMV infections [92].

#### 4.2.2. Studies in the Human Population

As mentioned before, HCMV infection is especially significant in immunocompromised patients, especially after transplantation. Therefore, studying the possible association between serum vitamin D levels and the risk of HCMV infection after transplantation is of great interest. However, it should be taken into account that most of these patients are treated with immunosuppression and viral prophylactic treatment, so the results are not usually extrapolated to the general population. In addition, results from studies in which the outcome definition was based on antibody titers must be considered critically due to the immunosuppressed state and the presence of comorbidities.

Most of the published studies were conducted on kidney transplant recipients (KTR). However, despite this common factor, there are still discrepancies regarding whether vitamin D serum levels can influence the risk of HCMV infections [93]. When vitamin D serum levels were measured only before [94] or up to four months after the transplant [78,95], the results showed no significant association between vitamin D status and the risk of HCMV infection. Nevertheless, when serum vitamin D levels were measured for six months or more, the results revealed a significant association between insufficient vitamin D serum levels and a major risk of HCMV infection [96]. The same result was observed in a study of neonates when the measurements were followed up to six months [97].

In addition, Bearden et al. observed that lower levels of maternal calcitriol and 25(OH)D in women infected with human immunodeficiency virus (HIV) are associated with a higher risk of congenital, perinatal, or postnatal transmission of HCMV [98]. Another study showed that vitamin D serum levels and HCMV antibodies were weakly correlated in patients with MS since children with sufficient vitamin D status had higher antibody levels against the virus than those with a deficiency [82].

Furthermore, there is also some evidence that may suggest that vitamin D supplementation may be associated with a minor risk of HCMV infection during transplantation, with vitamin D deficiency being related to a higher risk of HCMV disease. The study conducted by Moscarelli et al. observed a minor risk of HCMV infection in the group treated with calcitriol or analogues at least one month after transplantation, compared to the untreated group. Both the incidence and viral load were higher in the untreated group than in the calcitriol-treated group, although the duration of onset was paradoxically inverted [99]. All the studies concerning betaherpesviruses considered for this review and their characteristics are described in Table 2.

### 4.3. Gammaherpesvirus

The *Gammaherpesvirus* subfamily can infect epithelial and B cells, establishing latency in the latter. The main representatives of this subfamily are Epstein–Barr virus (EBV or HHV-4) and human herpesvirus 8 (HHV-8), also known as Kaposi’s sarcoma-associated herpesvirus (KSHV). EBV infection leads to mononucleosis in adult populations, and it has been associated with different neurological disorders, such as meningitis and encephalitis, as well as tumors like Burkitt’s lymphoma. On the other hand, HHV-8 infection causes Kaposi’s sarcoma in immunocompromised patients, and it is also associated with lymphoproliferative diseases and neurological syndromes. Both HHV-8 and EBV are mainly transmitted through saliva, although they may also be transmitted via blood transfusion and organ transplants [21,100,101].

#### 4.3.1. In Vitro Studies

There is some evidence that vitamin D and its derivatives can control the proliferation of Kaposi’s sarcoma cells and trigger autophagy in vitro, probably by downregulating different cellular pathways, such as PI3K/Akt/mTOR or NF-κB [102,103,104]. Thus, vitamin D may play a significant role as an immunomodulator in Kaposi’s sarcoma disease. It has also been observed that cathelicidin LL-37 can alter the envelope of HHV-8 by interacting with different glycoproteins on the surface of the virion, compromising the infection of oral epithelial cells. Since HHV-8 internalization appears to be more compromised than the attachment of the virus to the cells, LL-37 may exert its activity by binding to glycoproteins such as gB, which are essential for viral internalization [42].

EBV can cause lymphoproliferative disease in immunocompromised patients and is associated with Burkitt’s and Hodgkin’s lymphomas. EBV has also been considered a risk factor for MS in numerous reports, and a longitudinal study showed that individuals infected with EBV have a 30-fold increased risk of developing MS [105]. Most of the human population is seropositive for this virus; however, since the incidence of MS is not as high as EBV seroprevalence, there must be other risk factors that contribute to the onset of MS [106]. Several studies have shown a decrease in CD8+ T cells in patients with MS, while anti-Epstein–Barr virus nuclear antigen-1 (anti-EBNA-1 IgG) is increased [107]. Despite evidence that lower vitamin D levels are associated with an increased risk of MS [108,109], it is unclear whether this is related to EBV. In addition, in vitro studies have shown that EBV can use EBNA-2 and EBNA-3 transcriptional factors to modulate the vitamin D response as an immunomodulatory mechanism. Different researchers have proposed that both proteins may block the activation of vitamin D target genes, reducing vitamin D effects on immune cells [110,111]. However, it is uncertain whether this inhibition occurs through binding between VDR and EBNA-2 or through a competitive mechanism between the two factors to bind to the target genes. Regardless of the inhibitory pathways, this hypothesis is supported by the fact that these transcriptional factors and vitamin D have antagonistic effects on B-lymphocytes. While EBV uses EBNA-2 to induce cell growth in B cells, vitamin D enhances apoptosis [112,113].

#### 4.3.2. Studies in the Human Population

A prospective clinical trial performed in Zimbabwe found no association between HHV-8 viral load and vitamin D serum levels in patients with AIDS-associated Kaposi’s sarcoma [114]. However, this study only enrolled 90 participants; thus, further large-scale studies are still needed.

Regarding EBV, it is still not clear whether vitamin D supplementation affects EBV reactivation. On one hand, Rolf et al. studied the effects of vitamin D supplementation (14,000 IU/day) over 48 weeks compared to a placebo group in a randomized controlled trial with relapsing-remitting MS patients. The results showed no difference in EBV viral load or anti-EBV viral capsid antigen; however, anti-EBNA-1 levels were significantly reduced in the supplementation group compared to the placebo [115]. On the other hand, Zwart et al. studied the effects of vitamin D supplementation in Antarctic workers for 6 months at two different doses (2000 IU or 10,000 IU weekly). The results demonstrated a reduction in EBV shedding in saliva among participants with higher serum vitamin D levels [116]. Again, both studies have the limitation of a small enrolled cohort; therefore, it is not possible to establish a significant relationship.

Altogether, although there is no direct evidence of the effects of serum vitamin D levels on EBV reactivation, in vitro studies have shown that EBV has its own mechanisms to prevent vitamin D immunomodulation in immune cells, suggesting, in fact, that this molecule may have an immunomodulatory role in EBV infection [110,111]. All the studies of gammaherpesviruses considered for this review and their characteristics are described in Table 3.

## 5. Conclusions

Vitamin D has proven to be an important immunomodulator in chronic inflammatory diseases and infections since it can enhance self-tolerance and the release of numerous antimicrobial substances like cathelicidin LL-37, lysozymes, and defensins [4,41,56]. This antimicrobial activity has also been reported in other viral infections like hepatitis B infection [18,19].

However, the relationship between serum vitamin D levels and herpesvirus infections remains unclear due to several discrepancies in serum level classifications and vitamin D measurement. There is no consensus on which serum levels must be considered deficient or insufficient. Although most studies measure vitamin D serum levels as its major form (25-hydroxyvitamin-D) [88,96], some publications also measure the active form of vitamin D (1,25-hydroxyvitamin-D) [87,98]. Furthermore, many studies have overlooked key requirements for a nutrient intervention trial: the presence of vitamin D deficiency, often with unrealistically small sample sizes, and inconsistent intervention protocols regarding dosage and metabolites. The absence of standardized assays makes it difficult to interpret data from existing studies, which complicates the ability to combine results and conduct meta-analyses effectively. Additionally, results from studies carried out in hospital populations are rarely extrapolated to the general population due to the immunocompromised status of the cohort, especially when the outcome is defined by antibody titers. Furthermore, due to the high prevalence of herpesviruses like HSV-1, it is difficult to establish a relationship between vitamin D serum levels and HSV-1 infection [76].

Regarding vitamin D supplementation, some research attempting to link vitamin D levels with herpesvirus recurrences relies on information from clinical reports or self-reported questionnaires [88]. This reliance makes it difficult to establish a possible association, as it does not allow for adequate follow-up of patients and complicates the standardization of supplements. Therefore, further case–control studies are needed to determine whether vitamin D supplementation helps reduce herpesvirus reactivation. These studies will allow for proper comparison between the two study groups, enabling more extensive patient monitoring and reducing statistical interferences.

Despite the discrepancies in human population studies, it is worth noting that viral mechanisms to avoid vitamin D activity have been observed in many in vitro studies [91,92,110,111]. Further investigation is needed to determine whether these mechanisms also take place in in vivo models. For future studies, a standardized protocol for measuring and classifying serum vitamin D levels will be necessary. Since vitamin D supplementation is an affordable public health measure, exploring its connection to herpesviruses could offer a new strategy for reducing the health effects of these infections.

## Figures and Tables

**Table 1:** Characteristics of the Alphaherpesvirus studies in the human population.

Author, Year	Design	Viral Infection	Study Population	Intervention (Measurement)	Comparison/ Definition	Outcome Definition	Conclusion
Ranjbar et al., 2023 [86]	Cross- sectional study	HSV-1	Patients with recurrent herpes labialis (RHL) in the past 2 years (*n* = 85)	25(OH)D serum levels by ELISA KIT	Vitamin D serum levels compared to a control group	RHL recurrency in the last 2 years	No statistical differences between control and RHL patient serum levels
Mowry et al., 2011 [82]	Cross- sectional study	HSV-1 and HVS-2	Pediatric-onset multiple sclerosis (MS) (*n* = 140)	25(OH)D serum levels by chemiluminescent assay	25(OH)D insufficiency/ deficiency (<30 ng/mL)	HSV-1 and HSV-2 IgG serum titers measured via ELISA	Weak association between 25(OH)D deficiency and HSV-2 IgG viral titers
Chao et al., 2014 [87]	Case- control study	VZV	Patients undergoing maintenance hemodialysis for more than three months (*n* = 88)	25(OH)D and 1,25(OH)D serum levels by radio- immunoassay.	25(OH)D deficiency (<20 ng/mL) 25(OH)D insufficiency (20–30 ng/mL)	VZV-IgG and VZV- IgM serum titers	Statistical differences in VZV-IgG, but not in VZV-IgM serum titers
Lin et al., 2022 [88]	Cohort study	VZV	Patients of UK Biobank database (*n* = 177,572)	25(OH)D serum levels Self-reported Vitamin D supplementation	25(OH)D deficiency (<25 ng/mL) 25(OH)D insufficiency (25–49 ng/mL)	Clinical diagnosis of herpes zoster (HZ)	No statistical association between vitamin D supplementation and incident of HZ
Huang et al., 2022 [84]	Retrospective cohort study	HSV-1 and HSV-2	*n* = 14,174	25(OH)D serum levels measured via high- performance liquid chromatography- tandem mass spectrometry technique	25(OH)D deficiency (<20 ng/mL) 25(OH)D insufficiency (20–30 ng/mL)	Immunoglobulin G antibody titers of viral specific glycoproteins	Statistical association between vitamin D deficiency and anti-HSV-1/2 Ig-G titers
Öztekin et al., 2019 [85]	Case- control study	HSV-1	People with RHL and a volunteer group (*n* = 101)	25(OH)D serum levels	Comparison of vitamin D serum levels between people with RHL and control group	Clinical diagnosis	Association between Vitamin D serum levels and risk of RHL
Parvaie et al., 2021 [83]	Cross- sectional study	HSV-1	Dental students (*n* = 100)	25(OH)D serum levels	25(OH)D deficiency (<50 nmol/L) 25(OH)D insufficiency (50–75 nmol/L)	HSV-1 IgG serum titers measured via ELISA	No significant association between antibody titers and 25(OH)D serum levels

**Table 2:** Characteristics of the Betaherpesvirus studies in the human population.

Author, Year	Design	Viral Infection	Study Population	Intervention (Measurement)	Comparison/ Definition	Outcome Definition	Study’s Conclusion
Bearden et al., 2020 [98]	Retrospective cohort study	HCMV	Mothers with HIV and HCMV infections and their HIV- uninfected infants (*n* = 340 mother–infant pairs)	Maternal 25(OH)D serum levels measured via ELISA KIT during pregnancy Maternal 1,25(OH)D serum levels measured via ELISA KIT during pregnancy	25(OH)D insufficiency (21–31 ng/mL) 25(OH)D inadequate (11–20 ng/mL) 25(OH)D deficiency (<10 ng/mL)	HCMV testing via culture of urine and oral swabs and PCR studies of blood in infants	Statistical association between calcitriol levels and increased congenital HCMV infection
Astor et al., 2019 [96]	Retrospective cohort study	HCMV	Kidney transplant recipients (*n* = 1976)	25(OH)D serum levels at least 6 months after transplant via liquid chromatography	25(OH)D insufficiency (20–29 ng/mL) 25(OH)D deficiency (<20 ng/mL)	HCMV quantification via real-time PCR	Statistical association between 25(OH)D serum levels and HCMV infection
Mowry et al., 2011 [82]	Cross-sectional study	HCMV	Pediatric-onset multiple sclerosis (MS) (*n* = 140)	25(OH)D serum levels measured via chemilumuniscent assay	25(OH)D insufficiency/deficiency (<30 ng/mL)	HCMV IgG titers measured via ELISA	Statistical association between vitamin D serum levels and HCMV-IgG titers
Lee et al., 2014 [78]	Retrospective cohort study	HCMV	Kidney allograft recipients (*n* = 351)	25(OH)D measured within the first 30 days of transplantation	25(OH)D deficiency (<20 ng/mL)	Blood sample PCR or pp65 antigenemia	No statistical association between vitamin D deficiency and HCMV infection
Saber et al., 2015 [95]	Prospective cohort study	HCMV	Kidney transplant patients (*n* = 82)	25(OH)D measured within the four months after the transplant	Comparison of 25(OH)D serum levels depending on antibody anti-HCMV	HCMV-IgG and IgM measured	No statistical association between vitamin D serum levels and antibody titers
Park et al., 2017 [94]	Retrospective cohort study	HCMV	Kidney transplant patients (*n* = 164)	25(OH)D measured before the transplant via radioimmunoassay	25(OH)D deficiency (<20 ng/mL)	-	No statistical association between vitamin D deficiency and HCMV infection
Moscarelli et al., 2022 [99]	Retrospective cohort study	HCMV	Kidney transplant patients (*n* = 373)	Calcitriol supplementation (0.25 to 0.5 μg/day) at least 1 month before transplantation 1,25(OH)D serum levels measured via radioimmunoassay	1,25(OH)D deficiency (<20 pg/mL)	HCMV infection was defined as a blood viral load of >100,000 copies/mL	Statistical association between the incidence of HCMV infection and vitamin D supplementation
Wang et al., 2022 [97]	Prospective cohort study	HCMV	Full-term neonates (*n* = 471)	Measurement of 25(OH)D serum levels for 6 months	25(OH)D deficiency (<20 ng/mL) 25(OH)D insufficiency (20–30 ng/mL)	-	The incidence rate of HCMV infection was higher in the vitamin D insufficiency group

**Table 3:** Characteristics of the Gammaherpesvirus studies in the human population.

Author, Year	Design	Viral Infection	Study Population	Intervention (Measurement)	Comparison/ Definition	Outcome Definition	Study’s Conclusion
Rolf et al., 2018 [115]	Randomized controlled trial	EBV	Patients with relapsing-remitting multiple sclerosis (RRMS) (*n* = 53)	Vitamin D3 supplementation (14,000 IU/day) or placebo during 48 weeks Measurements of 25(OH)D serum levels	Comparison between supplementation group and placebo group of EBNA-1 IgG levels and viral load in EBV-specific CD8+ T cells	Analysis of EBV DNA PCR and HPRT gene expression via RT-qPCR Enzyme-like immunospot assay detecting EBV-specific activated CD8+ T cells EBNA-1, VCA and CMV IgG measured via ELISA	Statistical association between certain antibody titers and vitamin D supplementation
Zwart et al., 2011 [116]	Case–control study	EBV	People in Antarctica with no UV light exposure for 6 months (*n* = 41)	One group with 2000 IU/daily supplementation Another group with 10,000 IU/ weekly supplementation A group control without supplementation	25(Oh)D insufficiency (<20 ng/mL)	EBV viral shedding in saliva	Statistical association between 25(OH)D serum levels and EBV shedding in saliva
Mowry et al., 2011 [82]	Longitudinal cohort study	EBV	Pediatric-onset multiple sclerosis (MS) (*n* = 140)	25(OH)D serum levels measured via chemiluminiscent assay	25(OH)D insufficiency/deficiency (<30 ng/mL)	VCA and EBNA-1 IgG serum titers measured via ELISA	Higher Vitamin D levels were associated with higher antibody titers
Erlandson et al., 2014 [114]	Prospective clinical trial	HHV-8	People with AIDS- associated Kaposi’s sarcoma outcomes (*n* = 90)	25(OH)D serum levels measured via immunoluminometric direct assay	25(OH)D deficiency (<50 nmol/L) 25(OH)D insufficiency (50–75 nmol/L)	HHV-8 viral load in plasma	No significant association between HHV-8 viral load and vitamin D serum levels

## Abbreviations

1,25-(OH)2D3	Calcitriol
25(OH)D	Calcifediol/calcidiol
HSV-1/HHV1	Herpes simplex virus 1
HSV2/HHV2	Herpes simplex virus 2
VZV/HHV-3	Varicella zoster virus
HCMV/HHV-5	Human Cytomegalovirus
EBV/HHV-4	Epstein–Barr virus
KSHV/HHV-8	Human herpesvirus 8
VDR	Vitamin D receptor
MS	Multiple sclerosis
HIV	Human immunodeficiency virus
RHL	Recurrent Herpes Labialis
KTR	Kidney transplant recipient
EBNA	Epstein–Barr virus nuclear antigen
CIRDC	Canine infectious respiratory disease complex
SARS-CoV-2	Severe Acute Respiratory Syndrome Coronavirus 2
WHO	World Health Organization
CDC	Centers for Disease Control and Prevention
TLR	Toll-Like receptor
